# Effect of Remineralization Pretreatments on Human Dentin Permeability and Bond Strength

**DOI:** 10.1155/2023/2182651

**Published:** 2023-07-03

**Authors:** Amjad Abu Hasna, Frederico Canato Martinho, Pablo Lenin Benitez Sellan, Camila Reis Pampuri, Carlos Rocha Gomes Torres, Cesar Rogério Pucci

**Affiliations:** ^1^Department of Restorative Dentistry, Endodontics Division, Institute of Science and Technology, São Paulo State University – UNESP, São José dos Campos, São Paulo, Brazil; ^2^School of Dentistry, University of Maryland, Baltimore, USA; ^3^Department of Restorative Dentistry, Institute of Science and Technology, São Paulo State University – UNESP, São José dos Campos, São Paulo, Brazil; ^4^School of Dentistry, Universidad Espíritu Santo, Samborondón, Ecuador

## Abstract

This study aimed to evaluate Nd:YAG laser, calcium phosphate, and adhesive system effect as different pretreatments in different protocols on dentin permeability (DP) and bond strength (BS). Fifty human dentin discs were used (4 mm in diameter and 1,5 mm in height). Specimens were divided into five groups (*n* = 10): (A): adhesive system (control); (AL): adhesive system + Nd:YAG laser; (LAL): Nd:YAG laser + adhesive system + Nd:YAG laser; (PAL): calcium phosphate-based dentin desensitizer TeethMate + adhesive system + Nd:YAG laser; and group (PLAL): Nd:YAG laser + calcium phosphate-based dentin desensitizer + adhesive system + Nd:YAG laser. All materials were used according to the manufacturers' instructions. The specimens were submitted to artificial aging (5,000 thermal cycles and 12 × 10^4^ mechanical cycles) then a bond test was performed. DP was measured using the split chamber model. Data were submitted to one-way analysis of variance (ANOVA), paired *t*-test, RM ANOVA, and Tukey test (*p* < 0.05). All treatments were effective in DP reduction. For BS, the groups PAL and PLAL had improved BS with a statistically significant difference of the control group (A). Nd:Yag laser irradiation and calcium phosphate-based desensitizing agents significantly reduced DP, and the association between them could improve the BS on resin–human dentin interface.

## 1. Introduction

The adhesion of restorative materials–dental substrate interface has been improved by the acid-etching technique presented first by Buonocore [1], seeking out a more favorable surface for adhesion by chemical treatment to alter the dental substrate morphology. The acid-etching, as a choice, promotes the infiltration of monomers into the dental substrate to form a hybrid layer strengthened by the contact of collagen fibrils with the composite resin [2]. The hybrid layer has a principal role in the micromechanical retention and bonding process between composite resin and dentin. However, several factors can contribute to the breakdown of this adhesive interface. The hydrolytic and enzymatic degradation undermines with the adhesive interface and may initiate secondary caries over time [3]. However, despite the great advancement of adhesive materials over the years, this is a persistent problem.

Composite resin–dentin bond strength (BS) and biodegradation can be compromised by the presence of too much moisture [4]. For this, the laser irradiation of dentin surface, as an option, that results in the melting of the dentin and closure of exposed dentinal tubule orifices [5], may be indicated to obtain a less permeable dentin surface [6] and thus, less water presence that reduces the degradation of the adhesive interface [7].

Besides, some conditioning pretreatments of dentin can contribute to structural modification and structural rearrangement. The modification of temperature may cause water evaporation from the interaction layer, thus improving the dentin hardness and modulus of elasticity [8]. These changes in dentin can directly affect the permeability and durability of adhesive treatments. Many materials have been used with the purpose of reducing dentin permeability (DP) [9–11], such as the di- and tetra-calcium phosphate as effective materials in reducing DP and occluding the dentinal tubules that have superior performance compared to fluoridated varnishes [12].

The presence of calcium phosphate in combination with the laser can obliterate the dentinal tubules, reducing DP and decreasing the presence of water, which is one of the main problems related to the degradation of the adhesive interface. Therefore, the objective of this study was to investigate the effect of dentin pretreatments (Nd:Yag laser, calcium phosphate, and adhesive) on DP and composite resin–dentin BS. The null hypothesis of this study was that there would be no difference in DP and BS after applying the pretreatments.

## 2. Material and Methods

### 2.1. Experimental Design

**Figure 1:**
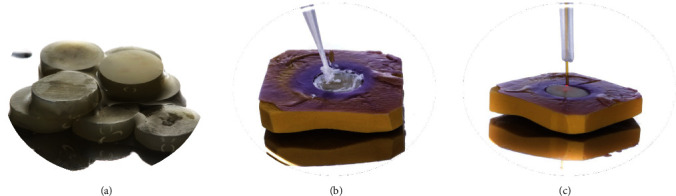
(a) Dentin discs, (b) calcium phosphate application, and (c) Nd:Yag laser application.

**Figure 2:**
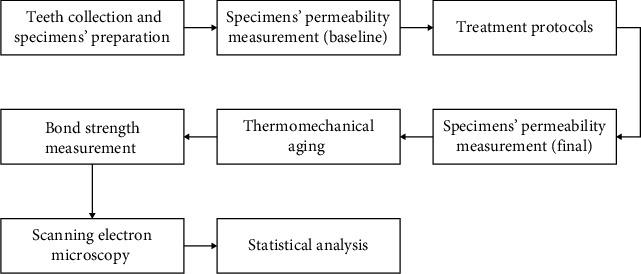
Flowchart of the methodology.

Fifty freshly extracted noncarious human third molars were collected based on a protocol approved by the Human Assurance Committee of the Institute of Science and Technology, Sao Paulo State University, Brazil, with informed consent obtained from the donating subjects with respect to the use of human tissues (N: 2.022.356). The research described has been carried out in accordance with the Code of Ethics of the World Medical Association Declaration of Helsinki for experiments involving humans. The extracted teeth were stored in distilled water at 4°C until the moment of use, not exceeding the period of 6 months (ISO 11405). Circular samples of dentinal disks (*n* = 10) were obtained of these molars with exactly 4 mm diameter and 1.5 mm thickness (Figure 1). The obtained samples were flattened and polished with abrasive paper 1,200, 2,400, and 4,000 at 300 rpm for 30 s of each one in a polishing machine (DP-10, Panambra, São Paulo, SP, Brazil) under water cooling to remove all excess enamel. The following flowchart explains the course of this study (Figure 2).

### 2.2. Permeability

The DP measurement was performed using the split chamber model through the THD-02c machine (Odeme Medical and Dental Equipment Ltda, Joaçaba, SC, Brazil). To measure the baseline DP of these samples, first, the formed smear layer on the pulp side of the disks was removed using 37% phosphoric acid for 15 s.

The rate of fluid movement in dentin was measured following the linear displacement of the air bubble into a capillary tube in which the water passes through a 0.01 mm resolution digital caliper. A baseline and final consecutive measurement of the bubble movement through the capillary were performed to compute the mean minimum DP values for each dentin disc. These measurements were represented by the distance traveled from the bubble in mm/min [9, 10].

To simulate dentin with open tubules, dentin surfaces were immersed in 0.3% citric acid for 30 s in an ultrasonic bath to remove the smear layer (Ultrasonic Cleaner 1440D, Odontobrás Indústria e Comércio, São Paulo, SP, Brazil) [11].

The maximum hydraulic conductance of each sample (Lpmax) was then obtained, representing the total opening of the dentinal tubules. After reading, they were immersed again in ultra-pure water (MEGApurity, Water Purification System, Billerica, USA) and ultrasonic washed for 10 min (Ultrasonic Cleaner 1440D, Odontobrás Indústria e Comércio, São Paulo, SP, Brazil).

The DP of each sample was calculated at each reading time after the respective treatments and proportionally as a percentage in relation to the maximum hydraulic conductance obtained after the opening of the tubules, which was considered a DP of 100% [9].

### 2.3. Treatment Groups

The specimens were divided into five groups (*n* = 10) according to the following:

- Adhesive group (A): only an adhesive system was applied.
- Adhesive and laser group (AL): adhesive system + Nd:YAG laser irradiation.
- Laser, adhesive, and laser group (LAL): Nd:YAG laser irradiation + adhesive system + Nd:YAG laser irradiation.
- Calcium phosphate, adhesive, and laser group (PAL): a calcium phosphate-based dentin desensitizer + adhesive system + Nd:YAG laser irradiation.
- Calcium phosphate, laser, adhesive, and laser group (PLAL): Nd:YAG laser irradiation + a calcium phosphate-based dentin desensitizer + adhesive system + Nd:YAG laser irradiation.

All materials (Table 1) were used according to the manufacturers' instructions.

### 2.4. Treatment Protocols

- The adhesive system: The Single Bond Universal adhesive system (3 M-ESPE, St Paul, Minnesota, USA) was actively applied in two layers with a microbrush for 20 s, followed by air drying for 20 s with a distance of 10 cm, and finally light-cured for 10 s (LED light, 1,200 mW/cm^2^, Radii-cal, Australia).
- The calcium phosphate-based dentin desensitizer: the TeethMate™ Desensitizer (Kuraray Noritake Dental Inc., Tokyo, Japan) was actively applied with a microbrush over samples for 30 s and then was washed and dried with absorbent paper.
- The laser irradiation: Nd:YAG Laser PulseMaster 600 IQ (American Dental Technologies Inc, Corpus Christi, Texas, USA) was applied at 1 mm distance perpendicularly to the sample surface with a wavelength of 1,096 *µ*m, noncontact at a pulse energy of 60 mJ/pulse, sweeping an average area of 4 mm in diameter for 60 s using the original quartz optical fiber (320 *µ*m in diameter and a pulse width of 0.1 ms) [12].

### 2.5. Bonding Procedures

After the application of the different adhesive techniques, two increments of 1.5 mm thickness of the nanoparticulate composite resin Filtek Z 350xT (3 M-ESPE, St Paul, Minnesota, USA) were added in all specimens. These were photoactivated for 20 s, at a standard distance, with the blue light-emitting LED light curing unit, wavelength ranging from 440–480 nm with a power density of 1,200 mW/cm^2^ (Radii-cal, SDI, Victoria, Australia).

### 2.6. Thermomechanical Aging

The specimens were submitted to thermomechanical cycling to simulate the 6-month aging in the oral cavity by means of the ER 37,000 thermomechanical machine (ERIOS Equipamentos Técnicos e Científicos Ltda., São Paulo, SP, Brazil). The specimens undergo 120,000 mechanical cycles and 5,000 thermal cycles with water baths for 30 s for each temperature (5 ± 2, 37 ± 2, and 55 ± 2°C) [13].

### 2.7. Bond Strength (BS)

This test was performed by the universal testing machine EMIC DL2000 with a 10 kg load cell. This machine has a coupled computer terminal, ready to read the data transmitted by the mechanical test through a program of its own. The specimens were fixed to a chemically activated transparent acrylic resin base with sticky wax. After fixation, the specimens were sectioned using an Isomet precision cutter (Buehler Ltd, Lake Bluff, IL, USA) under water irrigation on quadrangular section prisms, referred to as “sticks,” with approximate dimensions of 1 mm × 1 mm. Each specimen presented an average of eight “sticks,” whose area of the adhesive interface was measured with the digital caliper [14].

The sticks were individually fixed at their ends with cyanoacrylate glue (Zapit, Dental Ventures of American, Corona, CA, USA) into the metal microtensile device to perform the microtensile test on the test machine at a rate of 0.5 mm/min, at the time of fracture, the test was automatically stopped. All specimens that undergo pretest failures were discarded. The failure modes were evaluated at 50× magnification under a stereomicroscope (Stemi 2000C, Zeizz, Carl Zeis, Jena, Germany) and classified as resin, dentin, adhesive, or mixed failure [15].

### 2.8. Scanning Electron Microscope (SEM) Analysis

The dentin specimens of each group with adhesive or mixed failure and BS close to the mean of the respective group were selected for detailed analysis of dentin fracture in SEM. The specimens were placed in aluminum stubs, covered with gold/palladium (Desk II—Denton Vacuum), and examined in an SEM (Inspect S50 FEI, Czech Republic) operating with an increase of 2,000× and 5,000×.

### 2.9. Statistical Analysis

The DP data were analyzed by the one-way analysis of variance (ANOVA) and the *t*-test. The BS data (MPa) were analyzed by the repeated measure ANOVA and completed by the Tukey test, with (*α* = 5%) significance level.

## 3. Results

There was a significant statistical difference between the baseline and final DP readings in all experimental groups (Table 2), in which was observed a significant DP reduction. However, there was no significant difference when comparing the final DP readings among all groups *p* = 0.13.

For BS, in Table 3, there was no significant difference among the groups PAL, PLAL, AL, and LAL. However, the presence of phosphate was significant due to the statistical difference found between the groups with calcium phosphate (PAL and PLAL) and the control group (A).

**Figure 3:**
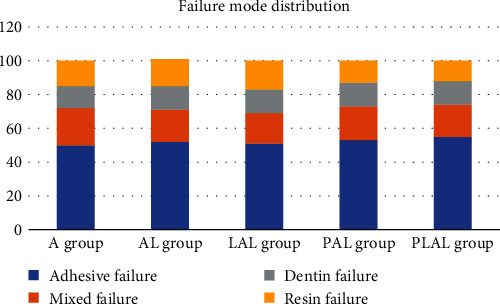
Failure mode distribution of all groups.

**Figure 4:**
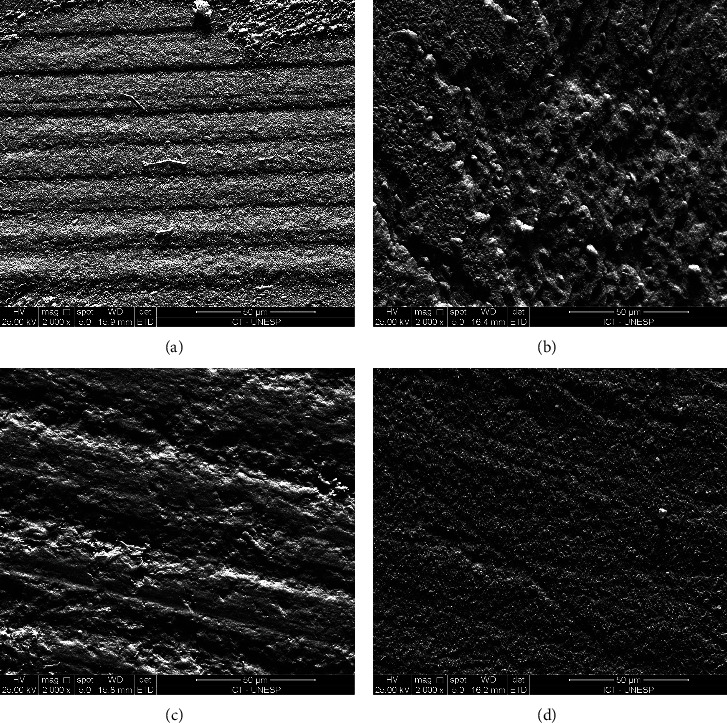
Scanning electron microscopy representative images of the dentin portion of the stick fractures: (a) PLAL group; (b) LAL group; (c) PAL group; (d) AL group.

Adhesive failure type was the dominant in all groups after the microtensile test, followed by the mixed type (Figure 3).  Figure 4 shows a dentinal portion of the fractured sticks of all experimental groups with the entry of the obliterated dentinal tubules.

## 4. Discussion

Dentistry has evolved well in recent years, simplifying operative steps with the development of various restorative materials. Among these materials, adhesive systems are composed of a variety of resinous monomers. Due to the advancement in the adhesion area, it is important to analyze the adhesive resistance and its behavior over time [16].

In this study, intact third molars were used to simulate in vivo conditions [17], which reflects results closer to clinical practice. For the DP test, dentin discs were used because of several advantages, such as ease of handling, good specimen finishing, and uniform thickness, compared to the crown segments, which by its pulp chamber structure, have varying dentin thickness [18]. Circular samples of dentin disks were obtained of these molars with exactly 4 mm diameter and 1.5 mm thickness due to difficulty in founding larger teeth as these used previously in the literature of 6 mm diameter [19].

The first part of this study aimed to evaluate the effects of Nd:YAG laser and calcium phosphate on DP. All experimental groups produced some degree of hydraulic conductance change. Our results show a significant reduction in DP when comparing the baseline and final readings of all experimental groups. This reduction may be due to the effect of the Nd:YAG laser because of its ability to enhance the amount of melting and recrystallization of dentin [20, 21]. This was found in the groups AL, LAL, PAL, and PLAL. Another study [22] revealed that the irradiation of dentin discs with Er:YAG or Nd:YAG laser is effective in reducing the DP.

Also, this DP reduction may be related to the presence of calcium phosphate in the desensitizer agent used in the group PAL and PLAL. Many studies in the literature agree with the results of our study in which the calcium phosphate-based desensitizer agents were effective in reducing DP [22–24].

In clinical practice, these procedures are indicated for hypersensitivity treatment, and many studies suggested the use of combined treatment for DP reduction by the association of laser irradiation and desensitizing agents and particularly calcium phosphate-based desensitizer [22, 25, 26] as the laser irradiation is considered safe and cause a minimal increase in the pulpal temperature, and consequently, not harmful to the pulp tissue as found previously [12], where the same parameters of Nd:YAG laser irradiation was used (60 mJ/pulse).

The moment of laser application seems to be a critical factor in DP, as found in the literature, once the laser applied prior to the adhesive system showed to be more effective in DP reduction [27]. However, another study [28] found a significant reduction in DP when the treatments were supplemented with laser. In the present study, laser irradiation in both before and after adhesive system application was effective in reducing DP, as found in Table 2.

Different materials have been released in the market to reduce DP, including silver-doped bioactive glass/mesoporous silica nanoparticle (Ag-BGN@MSN) [29] hydroxyapatite paste mixed with phosphoric acid [30] and fluoridated varnishes [31]. However, it was found that di- and tetra-calcium phosphate have a better performance in reducing DP and occluding dentinal tubules than fluoridated varnishes [32]. In this study, calcium phosphate was also effective in reducing DP.

The second part of this study was to evaluate the interface BS by microtensile test. The results showed no statistical difference among the groups AL, LAL, PAL, and PLAL. However, there was a significant difference when comparing the groups PAL and PLAL with the control group (A), and this may be explained by the association of both laser and calcium phosphate-based desensitizing agents. In the literature, the irradiation with Nd:YAG laser was effective in improving the adhesion between human dentin surface and self-adhesive resin cement [33] as it increases the resin infiltration into the dentinal tubules [34]. Besides, it improves the adhesion between lithium disilicate glass–ceramics, resin cement, and dentin [35]. However, it was revealed that the laser was not effective in improving the BS; these results agree with those found in the groups AL and LAL in which the presence of laser irradiation was not effective in improving the BS [36].

The use of desensitizing agents was effective in improving the BS of the adhesive system to human dentin [37] because it promotes dentin obliteration [38]; however, this is affected by the bonding agent used [39] and this may explain the negative results reported in the literature [40].

This is an in vitro study; therefore, the findings of this study should be evaluated clinically. In this study, we found that the combined effect of laser irradiation and calcium phosphate-based desensitizing agents were effective in improving the BS as the best results were obtained in PLAL group; however, the cost of laser should be considered.

## 5. Conclusions

The association of Nd:Yag laser and calcium phosphate-based desensitizing agents significantly reduced DP and improved the BS values on the composite resin–human dentin interface.

## Figures and Tables

**Table 1:** The materials used in the study.

Material	Brand	Manufacturer	Composition
Phosphoric acid	Magic Acid	Vigodent/Rio de Janeiro, RJ	37% phosphoric acid
Adhesive system	Single Bond Universal	3 M ESPE, St. Paul, MN, EUA	MDP, Bis-GMA, HEMA, diurethane dimethacrylate, polyalkenoic acid copolymer, camphorquinone, water, ethanol, glycerol, dimethacrylate, silica nanoparticles
Composite resin	Filtek-Z 350XT	3 M ESPE, St. Paul, MN, EUA	Bis-GMA, UDMA, TEGDMA, Bis-EMA, nanosilica filler, zirconia/silica particles
Nd:YAG laser	Pulse Master 600 IQ	American Dental Technology, USA	1,064 *μ*m wavelength and 320 m optical fiber
Desensitizing agent	TeethMate Desensitizer	Kuraray Notitake Dental Inc.	Powder: tetra-calcium phosphate, dicalcium phosphate anhydrous liquid: water, preservative

**Table 2:** One-way ANOVA test results and paired *t*-test for permeability.

Groups	Before treatment	After treatment	*T*-test^*∗∗*^
A	47.91 (±4.14)	26.76 (±3.37)	*p* = 0.0006 (*t* = 9.78)
AL	60.96 (±3.22)	35.20 (±2.95)	*p* = 0.0001 (*t* = 15.36)
LAL	58.83 (±7.01)	32.51 (±2.61)	*p* = 0.001 (*t* = 8.55)
PAL	47.39 (±9.79)	29.24 (±6.27)	*p* = 0.0.001 (*t* = 8.49)
PLAL	54.55 (±9.66)	32.93 (±11.47)	*p* = 0.005 (*t* = 5.45)
ANOVA one way		*p* = 0.13 (*f* = 1.69) ^*∗*^	

^*∗*^One-way ANOVA result for comparison between groups in the final assessment.  ^*∗∗*^Paired *t*-test for baseline and final comparison.

**Table 3:** Mean (MPa), SD, and homogenous groups of BS of each experimental group.

Treatment	Mean (Mpa)	±Standard deviation	Homogeneous groups
A	29.04	0.84	A
AL	29.49	2.15	AB
LAL	30.37	1.84	AB
PAL	31.08	1.04	B
PLAL	31.26	1.53	B

## Data Availability

The data that support the findings of this study are available from the corresponding author upon reasonable request.
